# The Pathogenesis of Tuberculosis: The Early Infiltrate of Post-primary (Adult Pulmonary) Tuberculosis: A Distinct Disease Entity

**DOI:** 10.3389/fimmu.2018.02108

**Published:** 2018-09-19

**Authors:** Robert L. Hunter

**Affiliations:** Department of Pathology and Laboratory Medicine, The University of Texas Health Science Center at Houston, Houston, TX, United States

**Keywords:** tuberculosis, pathogenesis, post-primary, human, x-ray, pathology, lung

## Abstract

It has long been recognized that tuberculosis (TB) induces both protective and tissue damaging immune responses. This paper reviews nearly two centuries of evidence that protection and tissue damage are mediated by separate disease entities in humans. Primary TB mediates protective immunity to disseminated infection while post-primary TB causes tissue damage that results in formation of cavities. Both are necessary for continued survival of Mycobacterium tuberculosis (MTB). Primary TB has been extensively studied in humans and animals. Post-primary TB, in contrast, is seldom recognized or studied. It begins as an asymptomatic early infiltrate that may resolve or progress by bronchogenic spread to caseous pneumonia that either fragments to produce cavities or is retained to produce post-primary granulomas and fibrocaseous disease. Primary and post-primary TB differ in typical age of onset, histopathology, organ distribution, x-ray appearance, genetic predisposition, immune status of the host, clinical course and susceptibility to protection by BCG. MTB is a highly successful human parasite because it produces both primary and post-primary TB as distinct disease entities in humans. No animal reproduces this sequence of lesions. Recognition of these facts immediately suggests plausible solutions, animal models and testable hypotheses to otherwise inaccessible questions of the immunity and pathogenesis of TB.

## Introduction

It the past 200 years tuberculosis (TB) has killed more people than all other epidemic infections combined and it still kills around 5,000 people per day, more than any other infection (1). Mycobacterium tuberculosis (MTB) is an extremely well adapted human parasite (2). While MTB can infect many animals, they cannot transmit the infection to others. The continued survival of MTB, therefore, depends upon transmission among humans. This is best accomplished by producing a cavity in the lung for proliferation of massive numbers of MTB to be coughed into the environment over a period of decades while the host remains healthy enough to circulate in the community. This requires that the host maintains effective systemic immunity to prevent disseminated infection by the masses of organisms being produced and released from the cavity.

While we are making previously unimaginable progress in defining the cells, molecules and pathways of TB, we are making little discernable progress in putting the pieces together to understand how the organisms accomplish the two functions of systemic immunity to protect the host and local susceptibility to produce and maintain a cavity. Our lack of understanding of these functions is still a major impediment to development of vaccines and new therapies (3–6). Dr. Anthony Fauci, Director of NIAID, expressed a nearly universal opinion with the statement: “We need to better understand the delicate balance between the host and pathogen in the context of the entire biological system and this requires a ‘radical and transformational approach.’ “Our goal should be to transform the entire field.” (7, 8).

## Shifting limitations of TB research

Inability to explain host resistance/susceptibility to TB is not a new concern, but the specifics have reversed. Fifty years ago Georges Canetti wrote “so much knowledge on TB mingles with so much obscurity on certain essential aspects of its pathogenesis … the obscurities are not due to lack of knowledge about the characteristic features of TB in man … The persistence of much ignorance in the pathogenesis of TB originates probably from lack of … experimental approaches to the disease” (9). Pinner expressed the same sentiment. “We understand the sequence of morbid changes that leads from infection to established pulmonary TB.” However, understanding the biologic processes “is a task for the years to come” (10). Canetti, Pinner and others knew the clinical presentation, pathology, and x-ray appearance of each of the stages of TB very well, but the basic science of their day was unable to address the biologic questions effectively. Today, the situation is reversed. We have the means to investigate disease in ways Canetti and Pinner could not have imagined, but we have forgotten the characteristic features of TB in man. Knowledge of the pathology of human pulmonary TB gained by 150 years of study by many investigators has been replaced by the fantasy that granulomas are the key lesion of all TB. Pinner wrote, “Progressive primary TB does not play a role in the development of phthisis in the adult” (10). Modern science is trying to understand the pathogenesis of TB in animals that don't develop the human disease guided by a badly flawed paradigm. Observations that do not fit with the prevailing paradigm are usually ignored.

The problem is that research on TB began around 1800, stopped in the 1950's and began again in the 1990s with little carry forward of information. The first phase in the preantibiotic era studied humans with untreated TB. Many detailed descriptions of the pathology, radiology and clinical course of untreated TB were published. The second phase continuing today uses advanced technologies to study animal tissues, human peripheral blood and BAL, occasional lymph node or other biopsies and lung resections of treated lesions, but not lungs with untreated TB. Unfortunately, none of these tissues contain the early infiltrate of post-primary disease. The called for “radical and transformational” approach may be simply to put the two phases together: to regain understanding of the pathology and clinical course of untreated human pulmonary TB and to use modern technologies to do coordinated studies of human and validated animal models to address the disease as it occurs in human lungs.

## Focused review of the literature on pathology of TB

Reviews have accused us of bias in review of the literature of the preantibiotic era. Indeed, the literature on TB has always been replete with disagreements, contradictions, and alternative hypotheses. Many studies focused on issues that are no longer pertinent. From the beginning, we focused on learning what investigators saw clinically and pathologically, not on how they thought it worked. Even so, finding understandable and consistent information in the vast literature on human pulmonary TB was a formidable task. The nomenclature had changed and the older papers contained few pictures. Little progress was made until we obtained histologic lung sections of acute post-primary TB and were able to see for ourselves the relevant lesions and understand the nomenclature. We have now collected histologic sections from over 100 autopsies of people with untreated pulmonary TB. This has produced a remarkable degree of consistency. In time, we focused largely on two questions: First, is there any support for the modern idea that cavities form by erosion of granulomas into bronchi? Second, does post-primary TB begin as an obstructive lobular pneumonia that spreads asymptomatically through bronchi before undergoing necrosis to become caseous pneumonia that fragments to form cavities? We were unable to find even a single article written by a person who studied the pathology of human TB that supports the former, but scores of publications that support the latter in whole or in part and none that contradict it.

## The first era of TB research (1800-1950)

**Figure 1:**
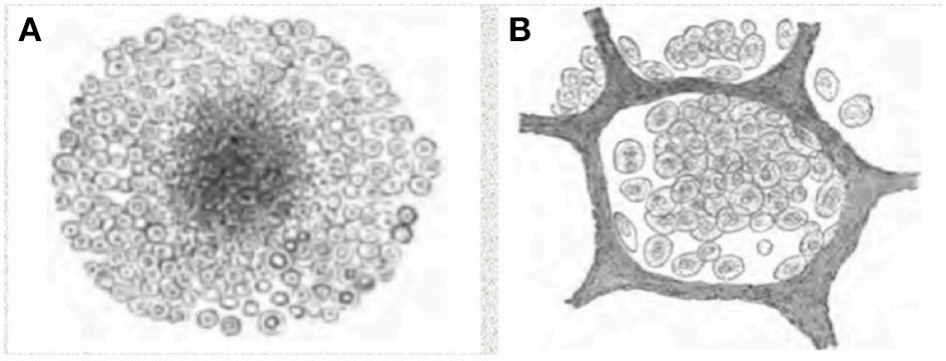
Characteristics of primary and post-primary TB. **(A)** Granuloma of primary TB, **(B)** Early alveolar infiltrate, Assmann's focus, of post-primary TB. Primary and post primary TB are distinct disease entities caused by the same organism, just as chicken pox and shingles are distinct disease entities caused by varicella zoster virus. Primary TB produces primary granulomas while post-primary TB begins with the early infiltrate. Over a period of months, most early infiltrates regress asymptomatically, but some spread via bronchi as obstructive lobular pneumonia, develop into caseous pneumonia that fragments to produce cavities or to become the focus of post-primary granulomas and fibrocaseous disease. The pathology of these lesions has been described since the 1850's and they have been monitored by x-ray since the 1920's. These drawings are from Cornil and Ranvier (14).

This era began when Laennec, using clinical observations, auscultation, and gross pathology, reported that 16 different conditions were all manifestations of one disease, TB (11). Microscopic observations begun in the mid nineteenth century demonstrated that granulomas were the characteristic lesion of primary TB, while obstructive lobular pneumonia was the characteristic lesion of developing post-primary disease (12). Pulmonary TB in the adult is different from primary TB from its inception (10). Rich wrote, “It has been found by all who have studied early human pulmonary lesions that they represent areas of caseous pneumonia rather than nodular tubercles” (13). The first demonstrable lesion of post-primary TB is a small focus of macrophages in alveoli, the early infiltrate, Figure 1 (10). This is a lobular pneumonia that spreads through bronchi, not the blood or lymphatics as does primary TB. It may regress or undergo necrosis to become caseous pneumonia that softens and fragments to produce cavities or is retained to become the focus of fibrocaseous disease (consumption or phthisis). Granulomas in post-primary TB form as a reaction to retained caseous pneumonia (15). Fibrocaseous disease begins as a post-primary granuloma that is easily distinguished from primary granulomas because its cores are composed of the ghosts of alveoli rather than being a homogeneous mass of caseous debris. Cavities arise from dissolution of caseous pneumonia, not from erosion of granulomas into bronchi as is commonly believed today. This is not a hypothesis or speculation, but is a scientific fact supported by dozens of publications by investigators who studied hundreds or thousands of cases of untreated TB over a period exceeding 150 years and it has been confirmed by modern investigations. (9–32).

Development of x-rays facilitated monitoring disease over time. In 1925, Assmann drew attention to the early infiltrate, a solitary infraclavicular opacity which he had observed by x-rays in young adults with slight symptoms, no physical signs and a definite history of contact with TB. He suggested that this opacity might represent the early tuberculous focus of adults (33, 34). The early infiltrate was considered important because it was the onset of TB in the age groups in which the disease did then, and still does, cause the greatest devastation (34). This was rapidly confirmed by other investigators (10, 35–37). One could frequently demonstrate that the early infiltrates were TB by culture of gastric aspirates. Worldwide interest in the significance of this early infiltrate, the Assmann's focus, stimulated numerous studies and publications through the 1940's (33–44) Investigators were able to longitudinally observe the progression and/or regression of subclinical post-primary TB for months before the onset of symptoms.

Studies of correlation of x-rays of the early infiltrate with pathological changes in the lung were conducted by multiple investigators (37). Typically, lungs were removed from the body, inflated to their normal size with formalin vapor and examined with stereoscopic x-ray plates. (43). The early infiltrates were shown to be small areas of exudative bronchopneumonic TB typically near the pleural surface in the upper posterior part of the lung. Few tubercle bacilli were seen by AFB staining. Using serial x-rays, it was noted that such lesions frequently resorbed as completely as pneumococcal pneumonias although much more slowly (10). The lesions pathologically were shown to be fan shaped with centered on small bronchi and extending to the pleura. The discharge of bacilli into the sputum might be only intermittent because of the semisolid caseous material produced bronchial obstruction that trapped the organisms (34).

Developing early infiltrates of TB seen on an x-ray plates were easily recognized as a diffuse heavy mottling, frequently seen scattered along the main trunks suggesting “raisins upon a stem” (43). In favorable cases, the opacity became less well defined, less homogeneous and less dense, and eventually completely disappeared (33). Such a focus did not give rise to abnormal physical signs, was often associated with no symptoms, either local or constitutional, but could be detected in a radiograph of good quality when it is less than a quarter of an inch in diameter (37). In unfavorable cases, the lesions became confluent and produced pseudo-lobar caseous pneumonia. Tuberculous broncho pneumonia could exist in a large area without causing any signs or symptoms. Its recognition suggests a grave prognosis. However, spontaneous regression with evident absorption of the exudate and clinical cure were well documented. In one report, noticeable absorption of tuberculous exudate occurred in 59 cases of a series of 489 or more than 12 percent. In 7 cases the change was slight, in 24 the change was moderate, in 18 the change was marked. There was clinical improvement all of the patients. By use of stereoscopic X-rays, the a most vital developmental phase of the disease can readily be followed and studied more fully in the living human. The observations were startling because the changes noted were neither slight nor infrequent (43).

A thorough study of x-ray chest plates made it possible read the changes in terms of actual pathology (43). This required coordination of the pathologic and x-ray examinations. The localized fans, cone-shaped lesions, walled in by the septa were never seen in post mortem studies if the pathologist's usual sweeping cut from apex to base was made. But careful dissection along the bronchi revealed these lesions immediately under the pleura as seen on x-ray. So, too, reading chest x-rays without respect to trunks also failed to see the characteristic fan shaped lesions. But again, by following different trunks, it was possible to interpret the images in terms of the actual progressive pathology (43).

The major x-ray findings of developing pulmonary TB were confirmed and extended with high resolution CT in 1993. Im reported that post-primary or reactivated pulmonary TB begins with an acute necrotizing pneumonia in the subapical lung, followed by transbronchial spread (38, 39). They also studied CT findings of pulmonary TB before and after treatment and correlated the results with isolated cadaveric lungs of patients who died of TB (38). The presence of multiple fluffy nodules approximately 5 mm in diameter, described as acinar nodules, was described as the classical radiographic pattern of bronchogenic dissemination of TB (38). Centrilobular branching linear structures, the tree-in-bud sign, were seen in all patients with newly diagnosed pulmonary TB or reactivation TB, except in a patient with miliary TB. These lesions disappeared after 5–9 months of treatment in all 40 patients. (38). The CT findings were superior to bacterial isolation from sputum in assessing response to treatment.

Several recent publications have confirmed and extended these findings and suggested that the two-state paradigm of active and latent TB is an oversimplification and that TB has as a spectrum of infection states, with a subclinical phase of disease during which pathology evolves before symptomatic presentation (45–49). The tree-in-bud sign is now recognized by some as a characteristic CT sign of developing post-primary TB. It is probably the same as the “raisins on a stem” pattern published in 1924, but with a much less well developed conception of the underlying pathology. New technologies have greatly expanded studies. For example, whole-blood transcriptional signatures have identified adolescents who were at risk of developing active TB up to 12 months before clinical diagnosis (22, 50). In addition, positron emission tomography and computed tomography (PET–CT) have been used to study both human and a animal TB with unprecedented precision (51). However, leading publications still state that “the pathological hallmark of human TB is the granuloma, which is an organized and localized aggregate of immune cells that consists of macrophages, lymphocytes and other host immune cells” (51). In our opinion, failure to recognize the true nature of the pathology of developing human pulmonary TB is a major implement to research.

The first period of research ended rather abruptly in the 1950's with the introduction of antibiotics and decline in availability of human tissues from autopsies. Many people thought that TB was no longer a problem. In addition, basic sciences of immunology and inflammation were diverging from morphologic pathology. As a result, much information was purposely ignored and rapidly forgotten.

It seems hard to believe that the scientific community could have forgotten key facts about the pathology, x-ray appearance and clinical presentation of TB that had been learned by over a century of research. However, when one considers the environment of the 1950', it becomes not only understandable, but arguably inevitable. Seaview Hospital in Staten Island NY is an instructive example (52). In 1940, Seaview had 1400 beds for TB patients and was the largest healthcare facility in New York. Physicians were very good at physical and X-ray examinations with pathologic correlations. According to their archives, “A ‘cure’ was discovered here in 1957.” The number of beds dropped to 26 and the hospital ceased operations (34). Most of the employees lost their jobs. In addition, the nation was recovering from two world wars and a depression. People wanted change. The “cure” was drugs that resulted from research in pharmacology, not pathology, or x-rays. Further, pathology and x-rays had little to contribute to the new sciences of genetics, cellular immunology, and macrophage biology that dominated the late twentieth century. Pathologists of the time were known to state that there was little as dull as a lung with a cavity (16).

## The second era of research on TB (1990-present)

Research on MTB never stopped, but the introduction of antibiotics shifted the focus away from the disease in humans to basic science topics. MTB was largely considered to be a model for studying basic aspects of immunology and inflammation. The threat of the disease, after all, had ended. Once a leading cause of death, TB was now widely considered relegated to history. Early studies focused on lymphocytes and macrophages and expanded to a broad spectrum of cellular and molecular sciences (53). Much basic immunology was learned from studies of purified components of MTB in animal models. This included the activation of macrophages in granulomas, new vaccine adjuvants, and immunomodulating agents. BCG and its components were intensively investigated as immunotherapy for cancers. Very few investigators had access to BSL3 facilities for animal infections.

The emphasis on human TB revived in the 1990's when increasing drug resistance and HIV stimulated renewed interest in the disease. By this time, investigators of the earlier period had retired, animal models for studies of basic immunology of granulomas were well established and most investigators, myself included, had never seen a case of TB. Since, MTB produced granulomas in animals, people studied granulomas (51, 54–56). Because of the decades long gap, established insights into the clinical course, pathology and x-rays of human pulmonary TB were forgotten and many misconceptions became dogma. There were multiple contributing factors. With the decline in autopsies and interested pathologists, investigators did not have either the knowledge or resources to challenge the emerging dogma. Our priority was to use new technologies to study basic biologic phenomena. The internet did not yet exist so it could not help with literature searches. Finally, driven by opinions of peer reviewers for journals and funding agencies, virtually the entire scientific world accepted the dogma that granulomas are the important lesion of all TB.

## Current paradigm of the pathogenesis of TB

Most recent research was guided by the paradigm that granulomas are the important lesion of both primary and post-primary TB. However, in over a decade of searching, we have not found a single article written by an investigator who personally studied the pathology of developing human post-primary pulmonary TB that supports the paradigm that granulomas are the characteristic lesion of both primary and post-primary TB and that cavities arise by erosion of granulomas in to bronchi. In other words, there is a disconnect between the clinical, x-ray and pathologic studies of the preantibiotic era and the basic understanding of the disease that guides today's research on TB. Some people dismiss the older reports as being “old,” not recognizing that they studied the actual human disease in ways that are no longer ethically possible. Furthermore, a paradigm is “a framework containing the basic assumptions, ways of thinking, and methodology that are commonly accepted by members of a scientific community.” (Dictionary.com). There is little chance of answering relevant questions if the assumptions, ways of thinking, and methodology discourage the most informative studies. Such paradigms are extremely difficult to dislodge (57).

The idea that cavities arise from erosion of granulomas into bronchi is a relatively recent concept. It became accepted as dogma during a period when cutting edge science used animal models and cell culture in the emerging fields of genetics, cellular immunology, and molecular microbiology. Morphologic pathology had little to contribute at this stage and was purposely ignored. TB produced granulomas in animal models and much excellent research was done to understand their biology. When interest was rekindled by the rise of drug resistance and HIV, investigators sought to justify their studies as models of human TB, but they had little knowledge of or access to untreated human tuberculous lung tissue. It had been reported that cavities in rabbits caused by *M. Bovis* form by erosion of granulomas into bronchi (58). It was, and is still, not generally recognized that MTB and *M. Bovis* produce cavities via different lesions (28). In the absence of contrary opinions, the observations using *M. Bovis* led to near universal acceptance of the paradigm that granulomas are the important lesion of both primary and post-primary TB. Guinea pigs are considered a good model because they produce “human like granulomas” without recognition that primary granulomas are only one type of human lesion. Similarly, mice are criticized because they do not produce caseating granulomas without recognition of the fact that their lesions resemble other phases of human TB (59, 60). Available evidence suggests that most, if not all, of the commonly used animal models develop lesions that are a mixture of elements of both primary and post-primary TB (60, 61). Most studies have focused on early events that are characteristic of primary TB. Enhanced recognition of the pathology of post-primary TB should enable design of animal experiments focused on that part of the human disease.

## Functions of primary and post-primary TB

**Figure 2:**
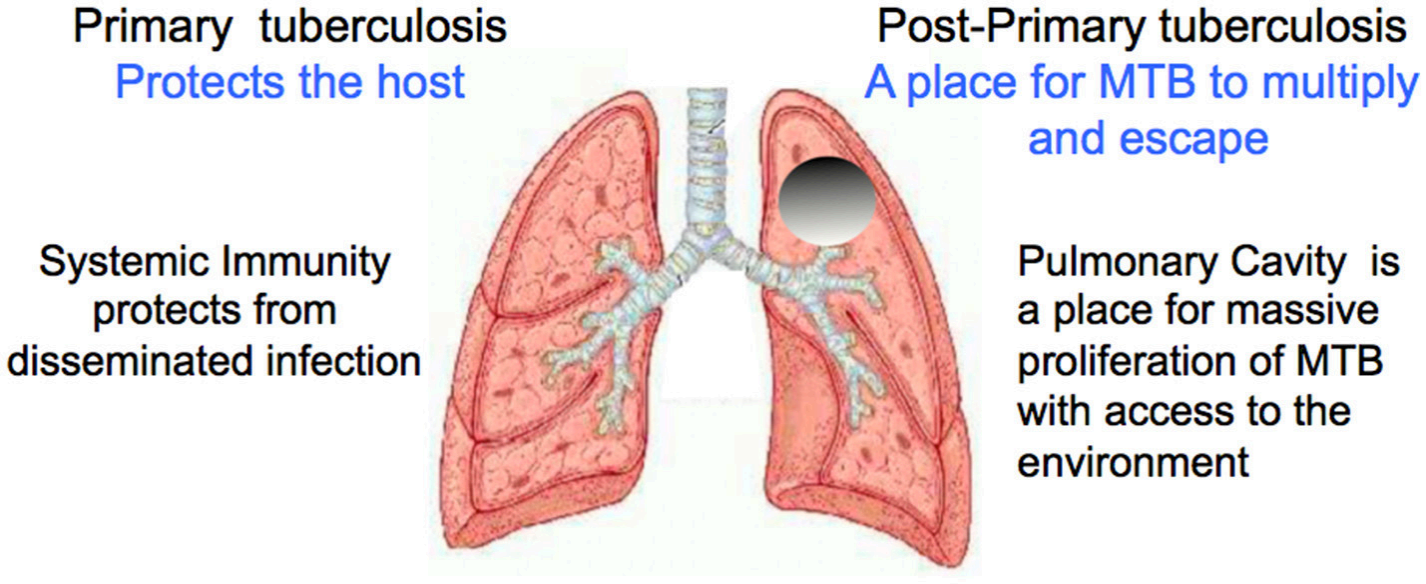
Functions of primary and post-primary TB. The survival of this parasite depends on keeping its host alive while large numbers of organisms are produced and transmitted into the environment over a period of decades. Image modified from https://www.slideshare.net/saktivinayaga/trachea-lungs Dr Mohammed Faez, Department of Anatomy IMS/MSU.

**Figure 3:**
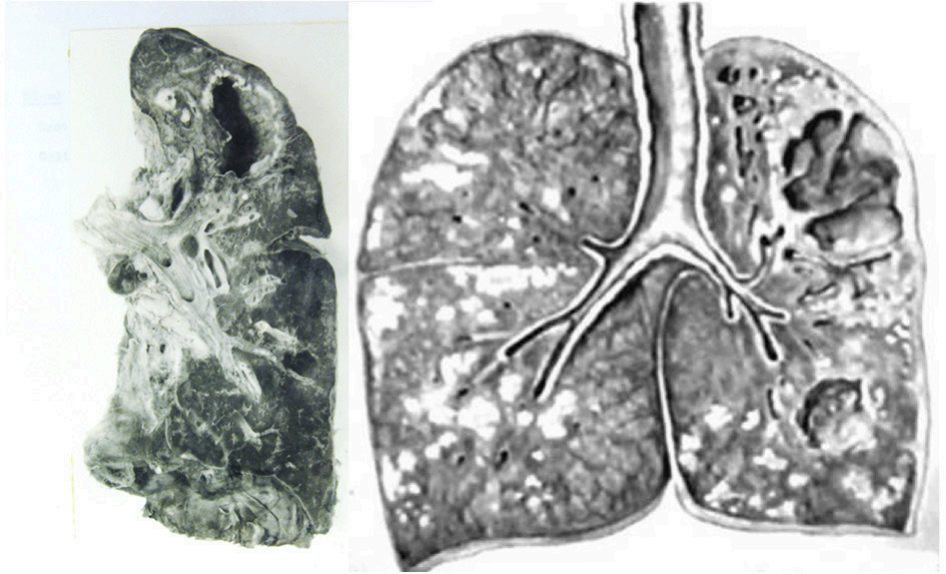
Gross images of pulmonary TB. **(Left)** Lung with cavity but no other active TB. This is the type of lesion is most favorable to the parasite because the person had only minor symptoms of TB and spread infection for years before dying of other causes. The entire host was protected except for the cavity. **(Right)** Lung of a person who died of post-primary TB. TB has continued to spread beyond the cavity as obstructive lobular pneumonia, caseous necrosis, and chronic fibrocaseous disease. The necrotic lung that was not coughed out was surrounded by granulomas to become the focus of post-primary granulomas. Photos courtesy of Rich (13).

The life cycle of MTB is to infect a person and induce systemic immunity that causes the lesions to heal. Then after a period that may extend for decades, develop an active pulmonary infection through either reactivation or reinfection that progresses to produce a cavity in the lung capable of producing masses MTB to be coughed into the environment. The organisms benefit most when large numbers of MTB pass through the airways and are expelled for decades from a healthy host, Figures 2, 3. Osler reported measurements of MTB in sputum of a man with moderately advanced pulmonary tuberculosis 16 times in a 3 month period. He found 1.5 to 4 billion organisms per 24 h (18). Illness or death of the host diminishes the chance of transmission of the parasite. Laennec reported people with large thin walled cavities lined by a gray membrane (pellicle of masses of MTB), but no other lesions of TB who had survived in good health for many years and died of unrelated causes (11). Such largely asymptomatic carriers of TB are well known to be especially dangerous for the transmission of infection (13, 18, 37). Accordingly, the survival of MTB depends on producing a cavity in which the organisms can divide in vast numbers in a host who is otherwise highly resistant to infection. These are the functions of post-primary and primary TB respectively. Primary TB protects the host by producing effective systemic immunity that prevents disseminated infection. Post-primary TB, in contrast, somehow evades and distorts systemic immunity to produce cavities in which the organism can multiply and escape into the environment. Primary and post-primary TB accomplish their functions through distinct pathologic entities.

Primary TB is the infection that occurs in a person with insufficient immunity to localize and control MTB in granulomas. It can produce a spectrum of clinical disease states ranging from disseminated TB in persons with AIDS, meningitis, miliary tuberculosis and probably extra pulmonary granulomas. It typically occurs in the very young, immunologically naive, very old, or immunosuppressed persons. The infection can disseminate via lymphatics or the blood stream to lymph nodes and diverse other organs. In immunocompetent people, the infection is controlled in weeks and the lesions heal. Primary TB produces systemic immunity that effectively protects the entire body from disseminated infection. This immunity has been extensively studied and has become the “central dogma” of protective immunity mediated by macrophages, granulomas and production of IFN gamma by CD4^+^ T cells (6). BCG replicates primary TB sufficiently to protect from disseminated TB and meningitis, but not from post-primary TB. Maintenance of protection depends on remaining immunocompetent. Lesions of primary TB can recur whenever immunosuppression reduces systemic T cell mediated immunity.

Post-primary TB, as its name suggests, typically begins only after primary TB has established systemic immunity. While the pathology and radiology of post-primary TB have been described in detail, very little is known about its mechanisms. The organisms somehow manipulate the host to produce the early infiltration. This isolates parts of the lung and so the organisms can survive in alveolar macrophages as an obstructive lobular pneumonia. Over a period of months secreted mycobacterial antigens and host lipids gradually accumulate in foamy alveolar macrophages that are trapped by obstructed bronchioles (26–30, 62). The early infiltrations slowly spread through bronchi to larger areas of lung. Many regress, but some undergo necrosis to produce caseous pneumonia that softens and is expelled to produce cavities or is retained to produce post-primary granulomas and fibrocaseous disease. Patients with the strongest immune responses measured by skin tests are the most likely to develop clinical disease (63, 64). This process was described by many pathologists in the pre antibiotic era and observed on x-rays by multiple investigators since the 1920's (33, 35). Several investigators reported that they could tell from x-rays who would develop clinical disease months in advance (35, 37). The radiologic appearance of the early infiltrate of subclinical pulmonary TB has been rediscovered several times. In the 1920's it was called Assmann's focus characterized by “raisin on a stem” appearance (33, 36, 43). In the 1990's, CT scans revealed greater detail and coined the term “tree-in-bud” sign as the characteristic feature of advancing pulmonary TB (65–67). In the past few years, this has been confirmed by studies in South Africa and Latvia using CT and PET-CT (22, 40, 45).

## Implications for future research

As discussed above, research on TB has already begun twice with little carry forward of information. Investigators in the first period developed detailed description of the pathology, imaging, and clinical presentation of untreated TB, but did not have the scientific tools to advance it. Investigators today have far advanced analytic tools, but are hampered by a flawed paradigm that does not recognize the sequence of lesions of post-primary TB. This is beginning to change with the increased recognition of subclinical TB and relevant animal models. Nevertheless, a third new beginning is now necessary to merge the clinical, pathologic and radiologic insights of the first era with the immunology, cell biology and genetics of the second so that the tools of modern science can finally be used to study the actual human disease. Recognition of primary and post-primary as separate pathologic entities immediately suggests resolution of long standing questions and multiple testable hypotheses as follows (28–30, 62, 68–70).

- **How can MTB be an obligate human parasite when people are more resistant than any of the animals studied?** Humans are more resistant to TB because most develop effective immunity against primary TB in weeks whereas most animals die within months of progressive disease composed of a mixture of primary and post-primary components. MTB is an obligate human parasite because only humans develop post primary TB that progresses to pulmonary cavities from which infection can be transmitted to new hosts.
- **What is the nature of the immunity that protects most people from post-primary TB**? Most current research focuses on control of primary TB by T cells and macrophages. Post-primary TB is different. It does not begin until primary TB has established a degree of systemic immunity. MTB apparently manipulates, strengthens, and uses our strongest immune responses locally in the early infiltration to develop caseous pneumonia and cavities from which it can escape to infect new hosts. Most early infiltrations regress spontaneously. The challenge is to understand why and develop means to make them all regress.
- **How can multiple pulmonary lesions in a single lung act independently as if the others did not exist?** Bronchial obstruction is a local process of the early infiltration that begins in different parts of the lung at different times. This starts the clock for accumulation of mycobacterial antigens, host lipids and cells that drive the disease.

## Obstacles to research

Several major obstacles must be overcome to pursue research on post-primary TB. The first is availability of informative tissue for study. Studies using peripheral blood, lymph nodes, and bronchoalveolar lavage cells are unlikely to be able to dissect multiple types of independently developing lesions in a single lung. Surgical resections and hospital autopsies seldom have the key lesions since nearly all people with TB who reach medical care are treated. As was discovered in the early days of antibiotic therapy, treatment rapidly abolishes the early infiltration of post-primary TB. The only sources are lungs from autopsies or emergency pneumonectomies for hemorrhage in people with untreated pulmonary TB. Patients who die of untreated TB are likely to be autopsied by medical examiners or forensic pathologists. Unfortunately few of these people work on TB or have close relationships with the TB research community. There are also significant legal, cultural and religious objections to research using autopsies. Nevertheless, with 5,000 deaths from TB/day, specimens do exist. Since human lungs frequently contain multiple lesions of TB that behave independently at both early and late stages, the entire range of disease processes could be studied in a small number of cases. The problem is to find them and build relationships with the appropriate people and institutions to access them for research.

Most widely used animal models are focused on early lesions in naïve animals that are largely models of primary TB. MTB is an obligate human parasite because no animal develops the post-primary lesions required for transmission to new hosts as do humans. However, many animal models can be adapted to produce lesions resembling those of particular stages of post-primary TB. For example, mice, rabbits, and guinea pigs all develop a pattern of pulmonary burden of MTB that is consistent with the early infiltrate of developing post-primary TB (60, 71). As stated by Robert North, a central problem in TB research is to explain why immunity to infection does not enable mice, guinea pigs, rabbits, or susceptible humans to resolve this lung infection and thereby stop the development of disease (61). Progressive pulmonary tuberculosis is not due to increasing numbers of viable bacilli in rabbits, mice, guinea pigs, and humans who develop paucibacillary disease, but is due to a continuous host response to mycobacterial products (72). This is reminiscent of the early infiltrate of post-primary TB in each of these species. Various manifestations of the primary and post-primary TB probably occur in animal models as described elsewhere, but they are not separated or coordinated as in humans (60, 71). This provides opportunities to develop animal models of particular components of post-primary TB by reproducing the conditions in animals that exist in humans at particular stages of infection (16, 36, 73).

## Potential of advanced technology

Driven largely by advances in cancer research, the capabilities for study of formalin-fixed, paraffin embedded (FPPE) tissues on slides has expanded enormously. In the preantibiotic era, pathologists could only look at routine H&E stained sections and count acid-fast bacilli. Now, multi-color immunofluorescence with advanced image analysis can be done on slides with preservation of the tissue architecture and intact microenvironment (74). Our preliminary studies illustrate the value of newer technology. Sections of characteristic lesions of human primary TB, caseating granulomas, early infiltrate and cavities of post-primary were selected for quantitative immunohistochemical studies of macrophages, lymphocytes, endothelial cells, and mycobacterial antigens (69). Abundant mycobacterial antigen, but very few AFB, were present in foamy alveolar macrophages of early infiltrates. Primary granulomas contained a preponderance of CD4^+^ T cells while the early infiltrate lesions contained more CD8^+^ T cells. More foxp3^+^ (Treg) cells were found in cavity walls than in other types of lesions. In other studies, we investigated the presence of regulatory markers associated within early infiltrates of post-primary TB (70). We chose three markers of mTOR signaling (pmTOR, insulin-like growth factor-1 receptor and activated Akt) and a second pathway of macrophage activation, COX-2. The results suggested that foamy macrophages in early infiltrate lesions over activate mTORC1, potentially inhibiting autophagy of the infected cell and limiting MTB killing. In addition, programed death-1 ligand (PD-L1) was highly expressed in foamy macrophages, surrounded by PD-1- expressing lymphocytes in the alveolar walls. Thus, in this critical MTB microenvironment of foamy alveolar macrophages, PD1, PD-L1 and two suppressor host response pathways appear active (mTOR and COX-2), possibly facilitating TB disease progression.

It is now possible to assess gene expression of both host cells and bacteria on routinely prepared slides (75). With recent advances in the immunotherapy of cancer, the methods for studying mutations, cell maturation and differentiation, immune parameters, inflammation, and healing on slides have advanced dramatically and will continue to increase (70). Thousands of studies can be done on single paraffin embedded samples over a period of years. Enhanced imaging can monitor the lesions over time. This is providing new opportunities for studying human tissues and for developing animal models to be used in a coordinated fashion with human tissues to successfully address previously inaccessible components of human TB.

In summary, evidence produced over nearly 200 years demonstrates that primary and post-primary TB are distinct disease entities that function to protect the human hosts from disseminated infection and to produce cavities for transmission to new hosts respectively. The existence of these two disease entities suggests plausible answers and testable hypotheses to long standing questions about the pathogenesis of TB and have significant implications for the design of vaccines and host directed therapies. However, post-primary TB is exceedingly difficult to study because it occurs fully developed only in human lungs and there is no ethical reason to do biopsies or resections of developing lesions. However, available evidence suggests that animal models can be constructed to replicate particular stages of the human post-primary TB. There is reason for optimism that coordinated studies of such animal models with available human tissues and advanced imaging will lead to significant advances.

## Author contributions

The author confirms being the sole contributor of this work and has approved it for publication.

### Conflict of interest statement

The author declares that the research was conducted in the absence of any commercial or financial relationships that could be construed as a potential conflict of interest.
